# Guidelines for the conduct of clinical trials in spinal cord injury: Neuroimaging biomarkers

**DOI:** 10.1038/s41393-019-0309-x

**Published:** 2019-07-02

**Authors:** Maryam Seif, Claudia AM Gandini Wheeler-Kingshott, Julien Cohen-Adad, Adam E Flanders, Patrick Freund

**Affiliations:** 1 Spinal Cord Injury Center, University Hospital Balgrist, University of Zurich, Zurich, Switzerland; 2 Faculty of Brain Sciences, Queen Square MS Centre, UCL Queen Square Institute of Neurology, London, United Kingdom; 3 Department of Brain and Behavioral Sciences, University of Pavia, Pavia, Italy; 4 Brain MRI 3T Mondino Research Center, IRCCS Mondino Foundation, Pavia, Italy; 5 NeuroPoly Lab, Institute of Biomedical Engineering, Polytechnique Montreal, Montreal, QC Canada; 6 Regional Spinal Cord Injury Center of the Delaware Valley, Department of Radiology, Division of Neuroradiology, Thomas Jefferson University, 1087 Main Building, 132 South 10th Street, Philadelphia, PA 19107 USA; 7 Department of Neurophysics, Max Planck Institute for Human Cognitive and Brain Sciences, Leipzig, Germany; 8 Department of Neurology, University Hospital Zurich, Zurich, Switzerland

**Keywords:** Spinal cord diseases, Predictive markers

## Abstract

Traumatic spinal cord injury (SCI) leads to immediate neuronal and axonal damage at the focal injury site and triggers secondary pathologic series of events resulting in sensorimotor and autonomic dysfunction below the level of injury. Although there is no cure for SCI, neuroprotective and regenerative therapies show promising results at the preclinical stage. There is a pressing need to develop non-invasive outcome measures that can indicate whether a candidate therapeutic agent or a cocktail of therapeutic agents are positively altering the underlying disease processes. Recent conventional MRI studies have quantified spinal cord lesion characteristics and elucidated their relationship between severity of injury to clinical impairment and recovery. Next to the quantification of the primary cord damage, quantitative MRI measures of spinal cord (rostrocaudally to the lesion site) and brain integrity have demonstrated progressive and specific neurodegeneration of afferent and efferent neuronal pathways. MRI could therefore play a key role to ultimately uncover the relationship between clinical impairment/recovery and injury-induced neurodegenerative changes in the spinal cord and brain. Moreover, neuroimaging biomarkers hold promises to improve clinical trial design and efficiency through better patient stratification. The purpose of this narrative review is therefore to propose a guideline of clinically available MRI sequences and their derived neuroimaging biomarkers that have the potential to assess tissue damage at the macro- and microstructural level after SCI. In this piece, we make a recommendation for the use of key MRI sequences—both conventional and advanced—for clinical work-up and clinical trials.

## Introduction

Spinal cord injury (SCI) leads to immediate and permanent neurological deficit below the level of injury as afferent and efferent neural traffic is disrupted [1, 2]. Rehabilitation can partially improve clinical outcome following SCI. Crucially, promising neuroprotective and regenerative therapies are imminent for neurological improvement [3, 4]. A combination of interventions (e.g. neuroprotective and neuroregenerative strategies) rather than one single treatment are needed to recover patient’s sensorimotor function both in the acute phase and in later stages [5]. At present, a number of active clinical trials are being conducted in SCI. The gold standard primary endpoint for SCI trials is based upon clinical assessment such as International Standards for Neurological Classification of Spinal Cord Injury (ISNCSCI) [6], which results in a large sample size and lengthy trial due to suboptimal patient stratification. Despite recent attempts to improve statistical approaches for predicting clinical endpoints and patient stratification [7], a large sample size is still required in clinical trials for demonstrating the efficacy of a therapeutic intervention using current clinical assessments. Moreover, these clinical assessments are insensitive to the underlying disease mechanisms in SCI and heterogeneous SCI subgroups [8]. An alternative strategy to track the therapeutic effects within short intervals (<24 months) and with practical sample size, is to use a standard outcome measure sensitive to underlying neurodegenerative processes that can track changes in the intended target of new interventions in clinical trials [5]. One way to establish a standard outcome measure is to supplement the current clinical assessments with non-invasive conventional and quantitative magnetic resonance imaging (qMRI) techniques [9]. Recent conventional MRI studies in spinal cord injury have shown great improvements in the detection and quantification of trauma-induced macrostructural changes at the focal injury site [10–13].

At the level of the injury, conventional MRI has been useful in determining the precise location and extent of intramedullary injury of an acute SCI outlining haemorrhages and oedema after human spinal injury. As oedema and haemorrhage subside, a post-traumatic cyst often appears alongside preserved tissue bridges detectable on MR images, and the magnitude of these changes predicts clinical outcome [14–16].

Although sensitive to pathology, conventional MRI lags to detect the quantification of trauma-induced disruption to the microstructure of the spinal cord because the signal intensity changes are poorly specific [17]. Quantitative neuroimaging techniques can overcome this limitation [18] by providing reproducible maps of “quantitative” values proportional to tissue microstructure such as myelin, axonal density, iron deposition, and metabolic profiling [19]. Recent qMRI studies in SCI showed secondary neurodegenerative changes occurring remotely in the cervical cord [2], in the lumbar cord [20] and in the brain [21–23]. qMRI measures of the spinal cord [17, 24] and brain integrity [25]—including atrophy, demyelination, and iron deposition—have demonstrated evolving and distinct markers of neurodegeneration that affect the entire spinal cord and brain [2, 22, 26, 27].

Advanced neuroimaging enables greater elucidation of the relationship between clinical impairment and abnormalities of the spinal cord and brain function [5]. Thus, qMRI measures can make a major contribution to the diagnostic work-up of SCI for recovery prediction and as a valuable outcome measure in clinical trials [27–29].

However, advanced neuroimaging methods have yet to be introduced into clinical practice and ultimately in clinical trials in SCI [24, 30]. The efforts need to be made to bridge this gap through proposing a consensus MRI protocol based on the standard and tested MRI sequences next to the specific post-processing software packages for all clinicians.

The aim of this narrative review is therefore to propose a guideline of clinically available MRI measures and the corresponding post-processing work-packages. We first lay out a summary of the main results obtained from established and advanced MRI methods in SCI which is followed by a discussion on the steps needed for their widespread adoption in SCI research and clinical trials.

## Conventional MRI of the spinal cord

### MRI assessment of the lesion site

**Fig. 1:**
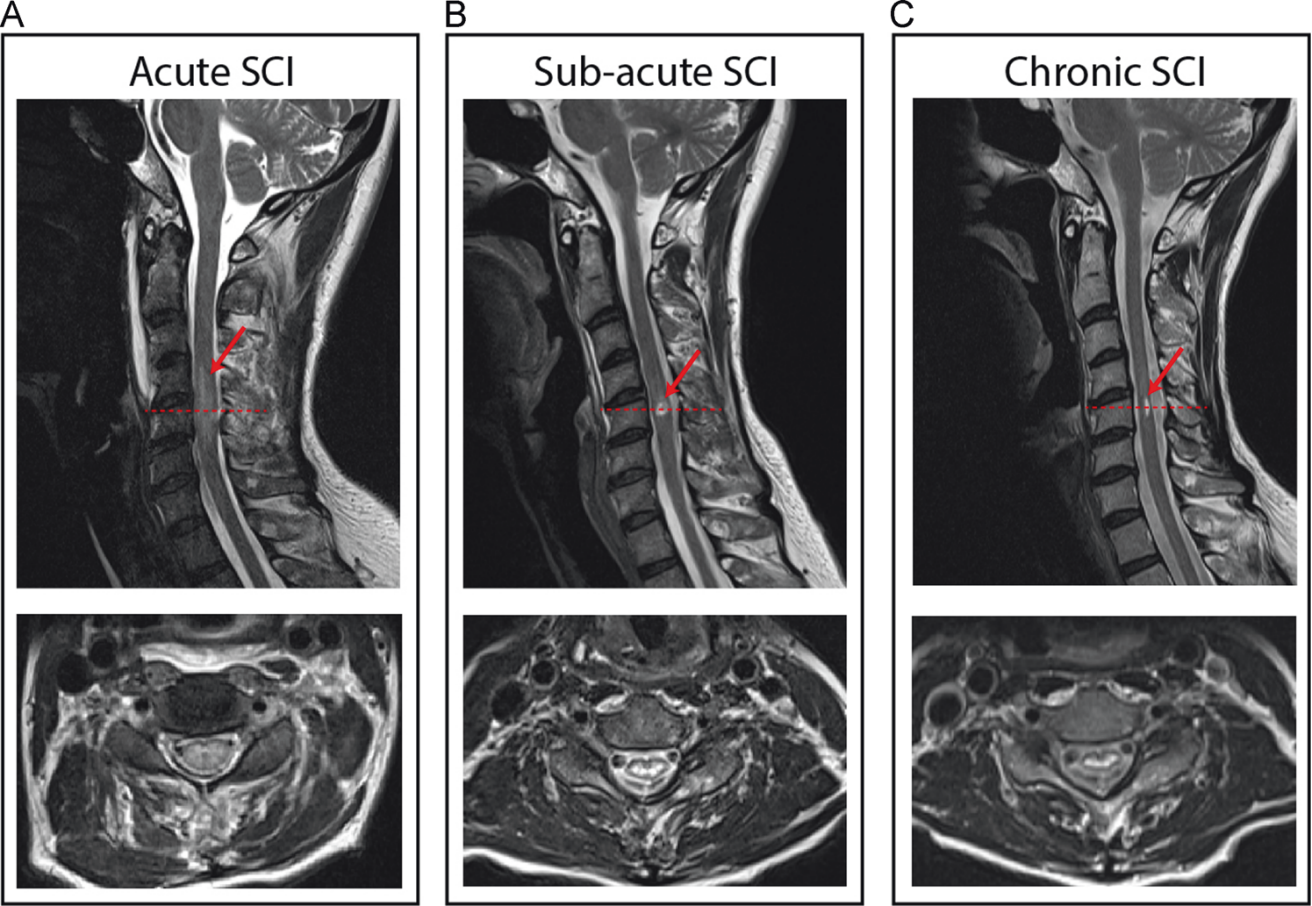
Overview of the lesion evolution with persisting midsagittal tissue bridges over time in a patient with traumatic cervical spinal cord injury. Sagittal and axial T2-weighted scans show the evolution of a cervical cord lesion from a 63-year-old  SCI patient (incomplete, female, AIS D) in **a** acute (1 day post-SCI), **b** subacute (1 month post-SCI), and **c** chronic phase (~24 months post-SCI)

Conventional MRI (T1- and T2-weighted scan) is routinely applied at the injury site in SCI to characterise the lesion in the acute phase, sub-acute and chronic SCI. This allows characterising the residual lesion structure (haemorrhage and oedema) and its extent early after trauma as well as elucidating the changes at the focal injury site over time [29] (Fig. 1). The length of the oedema and the size of haemorrhage at the lesion level is often associated with the initial neurologic deficit and recovery after injury [31]. The intramedullary lesion dynamically lengthens in the acute period, longitudinally enlarging nearly the height of one vertebral body without alteration in neurologic status [32]. In subacute SCI (1-month post-injury), the intramedullary lesion is remodelled [10], and a post-traumatic cyst develops [14–16]. The preserved tissue bridges can be identified in subacute phase dorsally and ventrally adjacent to the cyst on a T2-weighted MRI. Interestingly, the tissue bridges remain permissive for electrophysiological information flow and clinical recovery [14, 16]. Thus, when oedema and haemorrhage evolve in the subacute phase, the intramedullary lesion size is a good predictor of recovery. Conventional MRI scans at the lesion site show great potential to serve as neuroimaging biomarkers in clinical routine and in SCI trials as they are sensitive in detecting dynamic intramedullary signal alterations and preserved midsagittal tissue bridges in acute, sub-acute and chronic phases of SCI [14–16].

### Recommended conventional MRI protocol for scanning the lesion site

It is recommended to apply conventional MRI based on available standard sequences at the lesion site for characterising the intramedullary changes over time after injury. These product MRI sequences are sagittal and axial T1- and T2-weighted scans, which can be complemented with a short-T1 inversion recovery (STIR) sequence to assess the lesion site early after injury and following injury within clinical work-up and clinical trials. Gradient-echo T2* imaging (GRE) is useful for detecting microhemorrhages within the intramedullary lesion. T1- and T2-weighted MRI can also provide information on the extent and dynamics of midsagittal tissue bridges at the epicentre of the spinal cord lesion over time.

**Fig. 2:**
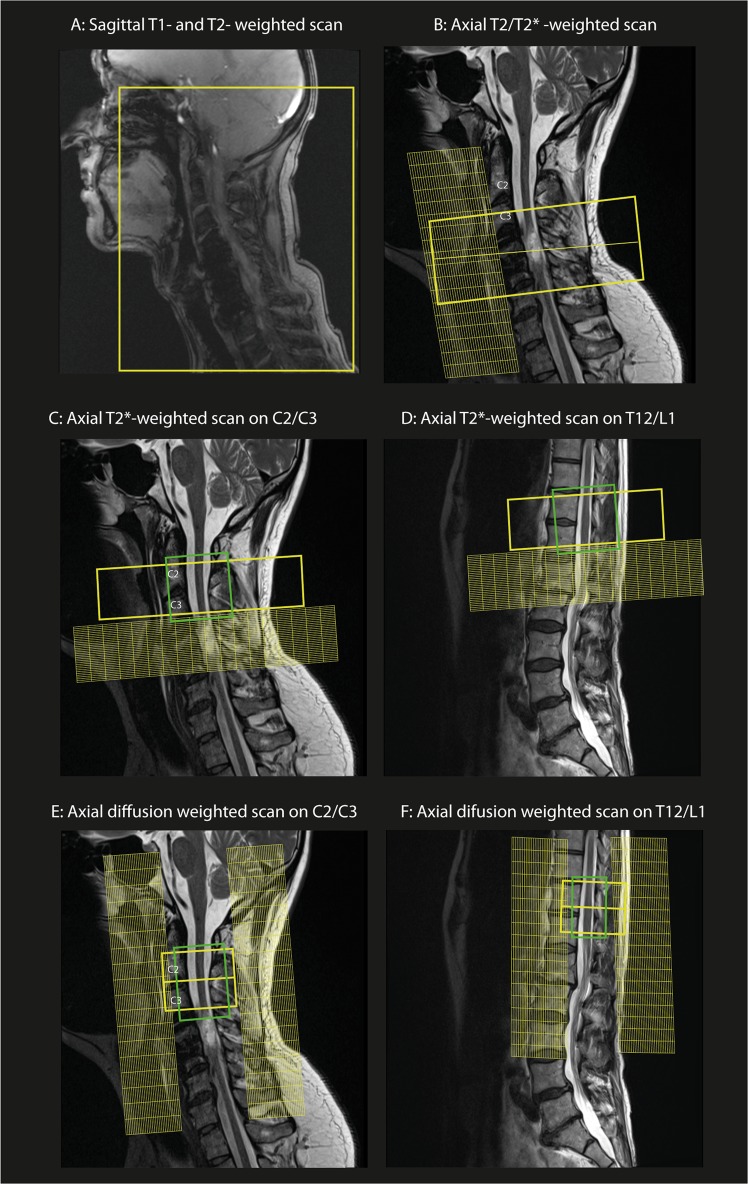
Spinal cord MRI protocol set-up: **a**, **b** Sagittal and axial T1-, T2- and T2*- weighted scans on the cervical spinal cord lesion. **c**, **d** Axial T2*-weighted scan set-up at the cervical and lumbar level above and below the lesion, respectively. **e**, **f** Diffusion MRI protocol set-up on the cervical spinal cord (C2/C3 level) and lumbar enlargement (T12/L1 level) with corresponding saturation bands

The field of view (FoV) of the cervical cord MRI protocol normally covers a portion of the posterior fossa until the upper thoracic spinal cord in the sagittal plane (Fig. 2a) while the FoV of thoracic MRI covers the entire length of the thoracic spinal cord. Axial T2-weighted-turbo spin echo sequence is normally placed on the lesion site perpendicular to the cord to assess the lesion and its axial extension (Fig. 2b). We suggest performing follow-up scans in clinical routine to track the lesion evolution after injury over time. The recommended MRI protocol is as follows:

- Sagittal T1- and T2-weighted-turbo spin echo sequence covering posterior fossa and the upper thoracic cord for tetraplegia or covering the entire thoracic spinal cord or lumbar spinal cord for paraplegia.
- Axial T2-weighted-turbo spin echo sequence placed on the lesion site perpendicular to the spinal cord.
- Sagittal and axial GRE T2*-weighted scan placed on the lesion site.

The sagittal T2-weighted scan is used for offline segmentation of the lesion area and axial T2-weighted MRI, helps users with navigating and positioning of the regions of interest. The total nominal acquisition time for the suggested clinical protocol (T1-w, T2-w, T2*-w scans) is ~12 min, although it may vary based on the MRI parameters of the sequence (e.g. image resolution, number of measurements, etc.) or the hardware design (e.g. the scanner vendor).

Interestingly the National Institute of Neurological Disorder and Stroke (NINDS) has the common mission of developing data standards and imaging protocols for the clinical research. The first set of Common Data Elements (CDE) features represents anatomic findings for the SCI that are routinely discernible on the conventional MRI.

### Data processing

To characterise the size of the cyst and the width of tissue bridges, the MRI data acquired from the lesion site can be segmented and then analysed using available software such as Jim software (Xinapse Systems, Aldwincle, UK) [14] or the Spinal Cord Toolbox (SCT) [33].

### MRI assessment above and below the lesion level

The majority of SCI patients undergo decompression surgery after injury and receive spinal metallic implants to manage spinal instability. Such devices cause significant MRI artifacts such as signal‐loss, geometric distortion, and failure of fat suppression, which worsen with increasing magnetic field strength. To reduce the severe artefacts due to surgical implants and to overcome issues linked to patient’s safety in the presence of such implants when using qMRI, trauma-induced structural changes are mainly assessed above and below the level of injury [34, 35]. Conventional MRI like T1-weighted and multi-gradient echo T2*-weighted scans provides images that can be used for the assessment of spinal cord atrophy by calculating the cross-sectional area of the cord above and below the injury level. Cord atrophy has already been postulated as a potential outcome measure in multiple sclerosis (MS) clinical trials of putative neuroprotective therapies [18]. Similarly, recent studies applying T1-weighted scans at the cervical spinal cord level in SCI showed, up to 7% (5 mm^2^) of atrophy within the first year following injury [21, 22]. Over time, cervical spinal cord atrophy continuously progresses to reach 14% at 2-year post injury [27] and up to 30% at 14 years after injury [36]. Thus, ongoing but slower cord atrophy is to be expected in chronic SCI over time. The magnitude of this atrophy was significantly associated with preserved electrophysiological and sensorimotor impairments in SCI [2, 20, 22, 27].

Recent studies have shown that the T2*-weighted scan allows a tissue-specific MRI assessment of the spinal cord (gray and white matter) above and below the lesion level (cervical and lumbar spinal cord, respectively) [2, 20]. For instance, a cervical SCI cohort showed signs of tissue-specific spinal cord atrophy in both the gray matter (GM) and white matter (WM) within the cervical spinal cord [2] and lumbar enlargement area in chronic phase [20]. These results indicate that the focal damage to the cord initiates neurodegenerative changes remote from the lesion affecting both WM and GM. Spinal cord atrophy measures should be considered in SCI clinical trials for tracking the potential effect of the treatments on the rate of atrophy (e.g. GM and WM cross-sectional area) as an outcome measure. To do that, it is necessary to apply a standard MRI protocol resulting in reproducible spinal cord volume metrics.

### Recommended MRI protocol for measuring spinal cord cross-sectional area

To assess spinal cord atrophy, a multi-gradient echo T2*-weighted scan (known as MEDIC on Siemens, mFFE on Philips, MERGE on GE scanner and ADAGE on Hitachi) can be used. These scans also provide excellent gray and white matter contrast. The FoV should be centred on the C2/C3 level in the cervical spinal cord (to assess above the lesion level), and on the T12/L1 level in the lumbar cord enlargement, perpendicular to the spinal cord to minimise partial volume effects and increase consistency between subjects (Fig. 2c, d).

Alternatively, isotropic resolution T1-weighted or T2-weighted scans, which are less prone to partial volume in the rostro-caudal direction, should also be used to compute spinal cord cross-sectional area (cord atrophy).

The conventional MRI protocol to asses cord atrophy therefore follows

- 3D sagittal T1-weighted MPRAGE sequence at 1 mm isotropic (minimum acquisition time: ~4 min) covering both head and cervical spinal cord. The T2*-weighted scan covering only the spinal cord (see below) is preferred.
- 3D sagittal T2-weighted at 0.8 mm isotropic (SPACE on Siemens, CUBE on GE, VISTA on Philips) (minimum acquisition time: ~5 min) placed on the C2/C3 level, or the T12/L1 level (lumbar enlargement) of the spinal cord.
- Axial T2*-weighted sequence (minimum acquisition time: 3.5 min) placed on the C2/C3 level or the T12/L1 level (lumbar enlargement) of the spinal cord.

Of note, for computing cord atrophy either the T1-weighted scan or T2*-weighted scan could be applied. However, we recommend T2*-weighted scans because they enable us to localize the WM and GM area and to calculate the tissue-specific cord atrophy. Recommended acquisition parameters for three main MR vendors, including standard operating procedure (SOP), are available on : http://www.spinalcordmri.org/protocols.

### Data processing

Offline calculation of the cross-sectional area metrics of the spinal cord can provide the rate of volumetric changes and tissue atrophy. Software packages to compute spinal cord and GM/WM area include: Jim software (Xinapse systems, Aldwincle, UK) for semi-automatic segmentation and SCT for automatic segmentation [33].

## Quantitative MRI and its benefits in spinal cord

### Diffusion magnetic resonance imaging of the spinal cord

Diffusion magnetic resonance imaging (dMRI) or diffusion weighted imaging (DWI) is a common qMRI method sensitive to the random movement of water molecules in tissues, applied in the brain and spinal cord. dMRI can reveal important microstructural properties of tissue like axonal structure and myelination [37]. The diffusion tensor (DT) model [38] is one of the computational diffusion models that can provide indices sensitive to tissue integrity. For instance, fractional anisotropy (FA) is mainly related to axonal count and myelin content [39], axial diffusivity (AD) and radial diffusivity (RD) reflect the integrity of axons and myelin, respectively [40]. Experimental evidence proved the association between DTI metrics and histological markers in-vivo, such as AD indicating axonal loss in a Wallerian degeneration model and RD indicating demyelination [41], although caution in this interpretation is needed [40]. Trauma-induced tissue changes at the microstructural level alter free water diffusion and can be quantified by the DT imaging (DTI) [42]. Thus, DTI has been applied to both cervical and lumbar cord to assess the severity of SCI and tract-specific microstructural changes in the spinal cord [2, 20]. DTI matrices have been reported to indicate anterograde and retrograde degeneration of sensorimotor tracts (decreased AD and FA), supporting the possibility of axonal loss both in rostral and caudal directions. These DTI outcomes were associated with clinical impairment and functional recovery in SCI [2, 20]. Such improved understanding of tissue-specific cord pathology offers potential biomarkers with more efficient targeting and monitoring of neuroregeneration (i.e., WM) in SCI. DTI therefore quantifies the degree of WM integrity, to predict recovery and to potentially monitor the effects of therapeutic interventions at the microstructural level.

### Recommended spinal cord dMRI protocol

Single-shot spin echo sequence with echo-planar imaging (EPI) readouts should be applied in the cervical cord with reduced FoV and the use of cardiac-gating to limit pulsation artefacts with about a 10-min acquisition time. The axial slices are orthogonally placed on the cervical cord (C2/C3 level) or the lumbar cord enlargement, (T12/L1 level) (Fig. 2e, f). There are typically 30 diffusion-encoded directions at *b* = 800 s/mm^2^ and 5  images with* b* = 0 (acquired at the beginning or interspersed of the measurement). To maximise signal to noise ratio (SNR), echo time (TE) should be minimised by using a monopolar gradient mode. A head-neck RF coil is preferable for the cervical spinal cord, while spine coils are used for the lumbar segments. Details of the recommended sequence parameters and SOP are available on: http://www.spinalcordmri.org/protocols.

### DWI data processing

**Fig. 3:**
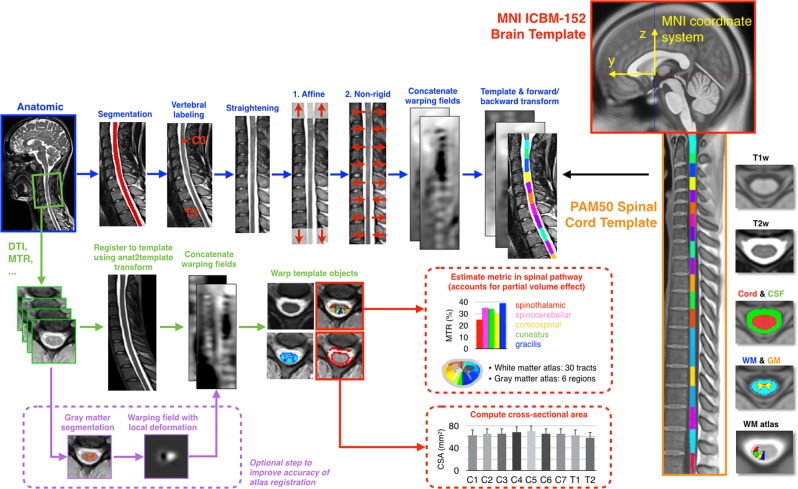
Overview of a template-based analysis pipeline using SCT: On the far right the PAM50 spinal cord template (orange box) and the MNI brain template (red box) are shown. First, anatomical data (e.g., T1-weighted or T2-weighted scans at 1 mm isotropic resolution or similar) is registered to the template (blue arrows). Additional quantitative MRI data acquired during the same scan session (e.g., DTI, magnetisation transfer, and fMRI) are registered to the anatomical data, and then template objects are warped to the multi-parametric data (green arrows). To improve accuracy of template registration, it is possible to add a step where the gray matter is segmented [66] and then warped to the gray matter template in order to update the warping fields (purple arrows). Subsequently, those MRI metrics can be quantified within specific WM tracts (red arrows). Cross-sectional area (CSA) of the spinal cord and gray matter can also be computed across vertebral levels. Adapted with permission [33]

qMRI data processing normally includes motion correction, registration to a spinal cord template, estimation of the qMRI metrics, and extraction of these metrics within template ROIs. SCT could be applied to process the spinal cord DWI data. SCT includes a spinal cord template (“PAM50”) [43], which is conveniently registered with the ICBM-152 MNI brain template, allowing simultaneous brain-spine studies. The PAM50 also includes a WM probabilistic atlas [44], and methods to extract metrics within specific WM tracts using Gaussian mixture method, which minimise the partial volume effect. Figure 3 illustrates an end-to-end processing of anatomical and DWI data for computing cross-sectional area (CSA) of spinal cord and DTI metrics in SCT. Alternatively, the combined brain and cervical cord template based in SPM12 can be used [45] to segment and spatially normalise brain and cervical cord MRI data (https://www.fil.ion.ucl.ac.uk/spm/toolbox/TPM/). Artefact correction could be performed with ACID toolbox (available on http://www.diffusiontools.com/) based in SPM12, which is an academic software toolkit for pre-processing diffusion MRI data, estimating DTI indices and perform spatial normalisation of DTI index maps (http://www.diffusiontools.com/).

### Myelin imaging of the spinal cord

Magnetic transfer imaging (MTI) is a qMRI method sensitive to myelin content [19] and to a lesser extent to axonal count [46] which has shown utility to study myelin integrity in SCI [26]. MTI typically uses an off-resonance pulse to excite protons bound to macromolecules, which have a wider resonance spectrum. These bound protons then transfer magnetisation to close-by “free” protons, effectively saturating the MR signal if imaging is performed shortly after MT. By acquiring one image without (MT0) and one image with a saturation pulse (MT1), one can compute the MT ratio (MTR) which, correlates with myelin concentration in tissue because of the macromolecular structure of lipids in myelin sheath [17]. However, MTR is not an absolute measure of myelin content and is notably affected by T1 and B1 field variations. To mitigate this limitation, MT saturation (MTsat) is suggested in which a third image is acquired with a shorter repetition time (TR) and/or higher flip angle, to fit the T1 component [46]. MTsat is notably implemented in the so-called multi-parameter mapping (MPM) method [47]. The MPM method is composed of three different multi-echo 3D fast low-angle shot (FLASH) gradient-echo sequences, designed to provide MR parameter measures of longitudinal relaxation rate (*R*1 = 1/T1), effective proton density (PD*), magnetisation transfer saturation (MTsat) and effective transverse relaxation rate (*R*2* = 1/T2*) to assess microstructural changes associated with myelin and iron content in the brain and cervical spinal cord. Previous studies reported that MTI readouts are strongly correlated with the histologically measured myelin content [17, 48] making MTI an attractive tool to assess myelin integrity in different pathologies including MS [49], Alzheimer’s disease [50] and SCI [22, 26]. The MPM protocol has been used to assess microstructural changes induced by injury in the spinal cord [22, 26]. The MPM maps revealed that in areas undergoing progressive atrophy, myelin content decreased and iron content increased [21, 22, 26, 27]. These bidirectional changes are suggestive of demyelination and iron accumulation, both processes known to occur during neurodegeneration after SCI [27]. Currently, the MPM approach (based on vendor’s sequences) is employed in a multi-centre study (INSPIRED) to understand mechanisms of myelopathy and in a European multi-centre clinical trial (NISCI) [51] as an exploratory outcome to investigate treatment effects of an anti Nogo-A antibody drug which facilitates regenerative sprouting after injury [52]. MTI holds the potential in SCI for an outcome measure in clinical research as MT maps can be predictors of recovery.

### Recommended spinal cord myelin imaging protocol

To calculate MTR, a protocol consisting of two 3D spoiled gradient-echo sequences, one with MT pre-pulse (MT1) and one without MT pre-pulse (MT0) is required combined with a T1-weighted sequence as a reference image. The MT protocol covers the cervical cord (above the lesion) using the head-neck RF coil available with most clinical scanners. To calculate MTsat, a third gradient-echo sequence can be added, more sensitive to T1-weighted effect, hence with shorter TR and/or higher flip angle. Recommended sequence parameters and SOP are available at: http://www.spinalcordmri.org/protocols.

The MPM protocol consists of three multi-echo 3D FLASH sequences, run with different TR and flip angle to obtain T1-, MT-, and PD-weighted images with 1 mm isotropic resolution. The FoV (240 × 256 mm^2^) can be applied to full brain and cervical cord down to the C5 level, or only the cord level with reduced overall coverage using the head-neck RF coil. The MPM protocol and corresponding SOP are available on http://hmri.info.

###  MTI data processing 

Analogously to the processing of DWI data, the method here consists of computing myelin-sensitive metrics (e.g., MTR, MTsat, T1, MPM), registering those metrics to a template, and extracting those metrics using an atlas-based approach. Like mentioned above, SCT or the combined brain and cervical cord template [53] available in hMRI toolbox should be used.

## Potential neuroimaging biomarkers in brain

### Brain atrophy measures

Brain volume changes measured by MRI are well-established end points of clinical trials in MS [54, 55]. Volumetric MRI measures have also been suggested as outcome measures in clinical trials assessing disease-modifying therapies in Alzheimer’s disease [56, 57]. In acute and chronic SCI, cross-sectional and longitudinal measures of the brain volume, as per voxel-based morphometry analysis [58], have shown correlation with neurological deficits after SCI [22, 26, 27, 59].

A recent longitudinal study [27] that acquired T1-weighted images over two years in six time-points reported significant WM volume reduction within the corticospinal tract (CST) of SCI compared to healthy controls. Outside the CST, WM volume decreased in the medulla oblongata and cerebellar vermis. GM volume decreased in the left insula, left ACC, and right thalamus. Most of these atrophic changes have shown significant associations with clinical outcomes in SCI patients. These measurable changes are sufficiently large, and predictive to consider them as sensitive outcome measures for clinical trials and assess the effect of experimental agents on atrophy rate in brain specific regions.

### Brain microstructural measures using qMRI

Brain DTI applied in SCI cohorts showed a reduced FA, reflecting microstructural tissue changes within the CST [60, 61], and centrum semiovale [62] compared to healthy controls, which may be a complementary predictor of fine motor damage and recovery. DTI therefore promises to quantify the degree of WM integrity, to predict recovery, and to potentially monitor the effects of therapeutic interventions in SCI.

Previously, SCI-induced microstructural changes have been investigated in the cervical cord and brain using MPM technique [22, 26, 27]. These studies reported myelin decrease and progressive structural changes along the neuroaxis following trauma in acute [21] and chronic SCI patients [26, 27]. Crucially, myelin-sensitive MRI parameters (R1 and MT) at 12 months were reduced within, but also beyond, atrophic areas [22]. These findings may be the results of retrograde degeneration of myelinated axons following SCI. Assessment of the underlying pathophysiology, with a myelin-sensitive MPM approach, supports the assumption that the volume changes in the brain relate to atrophy of myelinated axons and their cell bodies within the gray matter of sensorimotor cortices [63]. Based on the qMRI longitudinal studies in SCI and from a clinical perspective, a notable association exists between neurodegeneration changes in the brain and the magnitude of the recovery in patients. The finding of a systematic degenerative pattern with time suggests that non-invasive MRI measures could be used for prediction of outcome, identification of patients most likely to benefit from different interventions, and as potential markers of treatment effects of interventions (physical, drug, or cell-based therapies) [22].

### Recommended brain MRI protocol

#### T1-weighted brain scan

3D T1-weighted scans covering the full brain, including brainstem and cerebellum, and extending down to the cervical cord are required to assess local volume changes in the brain and spinal cord (i.e. applying voxel-based morphometry (VBM) processing method). Different acronyms exist with different sequence implementations aimed at obtaining 3D T1-weighting: FSPGR (GE), 3D TFE (Philips), MPRAGE (Siemens and Hitachi), 3D FFE (Toshiba).

#### Data processing

To calculate local volume changes, T1-weighted scans  could be processed with the VBM toolbox (Ashburner and Friston 2000) implemented in SPM12 (https://www.fil.ion.ucl.ac.uk/spm/).

#### Diffusion weighted scan

Whole-brain dMRI can be acquired using a single or double spin echo sequence with 60 diffusion weighted images (*b*-value = 1200 s/mm^2^) and 7 T2-weighted images (*b* = 0 s/mm^2^) with voxel size = 2.5 × 2.5 × 2.5 or less if time and signal to noise ratio allow it. The acquisition time for dMRI is ~8 min, depending on subject’s heart rate if cardiac gated.

#### Data processing

There are several software packages for DTI analysis of dMRI data, including FDT (FMRIB’s Diffusion Toolbox) in FSL [64] and TRActs Constrained by UnderLying Anatomy (TRACULA) toolbox [65] based on FreeSurfer software, and ACID toolbox based on SPM12. A reference structural image (e.g. the 3D T1-weighted scan suggested above for atrophy) should be acquired for DWI brain registration to other modalities like MPM or to a brain template.

####  Myelin imaging

As explained earlier, the MPM protocol consists of 3 multi-echo 3D FLASH sequences with different weightings, which are applied to the brain and spinal cord to obtain MT, PD and T1-weighted images. The FLASH sequence is available on all vendors. However, on Philips scanners an extra research software package is needed to set up more than 5 echoes for the multi-echo sequences. The currently established MPM protocol is not available on GE scanners due to a sequence limitation on the number of possible echoes. However, work in progress aims to provide full compatible protocols on all types of vendors. Of note, the MPM protocol could be potentially set up with a lower number of echoes at the price of lower maps resolution and SNR.

#### Data processing

The hMRI toolbox [45] is available to calculate the MPM maps with a detailed manual of how to process the MPM data (http://hmri.info).

### Sample size calculations based on qMRI measures

**Fig. 4:**
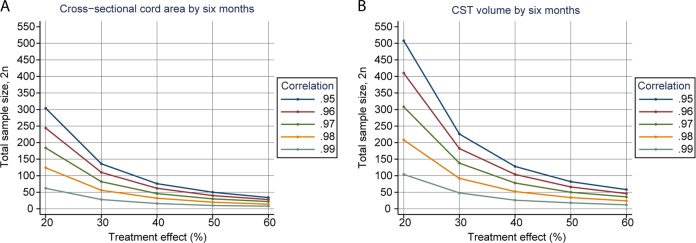
The relationship between the sample size required for a 6-month randomised clinical trial to detect a 100% treatment effect with 80% statistical power and 5% significant differences between healthy controls and patient group for **a** cross-sectional cord area and **b** cranial corticospinal tract (CST) volume. The calculations cover a range of plausible baseline vs. 6-month correlations around the observed patient values of 0.99

There is an imperative to improve clinical trial design by optimising patient stratification in the context of disease heterogeneity in SCI. In previous studies employing qMRI [23] concomitant longitudinal effect sizes have been estimated based on the sub-acute qMRI for clinical trials by means of power calculations. Figure 4 shows estimates of sample sizes for trials expected to enrol patients receiving treatment over 6 months, assuming two groups of randomised clinical trial arms with MRI outcomes of (a) spinal cord cross-sectional  area, (b) CST volume, to detect treatment effects with 80% power at 5% significance. The calculations cover a range of plausible baseline versus 6-month correlations around the empirical patient values of 0.98 for the spinal cord area and 0.99 for CST volume. The correlation refers to the correlation coefficient between baseline clinical measures and follow-up clinical measures: the higher this correlation the more random error explained by the baseline value and so the greater the power and smaller the sample size. For example, the required sample size for a 30% treatment effect (i.e. slowing of the rate of change on MRI readouts) assuming a correlation coefficient of 0.98 for the cord area and 0.99 for the CST volume, is 26 and 25 subjects per arm, respectively. Thus, if the estimated longitudinal effect sizes, as a function of post-trauma time, are substantial as indicated by the calculations, the possibility of quantifying responses to therapeutic interventions within 6 months after injury is exciting. In short, qMRI biomarkers of neurodegeneration represent promising instruments for the stratification of patient cohorts and the improvement of trial efficiency. However, it is imperative that we await results from ongoing clinical trials that have already included such qMRI measures as exploratory outcomes.

## Conclusion

This narrative review proposes a guideline of clinically available MRI measures that have the potential to assess tissue damage and repair at the macro- and microstructural level after SCI which may be applicable to clinical trials and diagnostic work-up. The combination of serial conventional MRI and qMRI with clinical outcomes represents a feasible mean for a better evaluation of complex changes following SCI and for uncovering the intricate relationship between clinical impairment and primary and  secondary remote neural changes in the uninjured spinal cord and brain. Furthermore, these quantifiable changes appear to have notable predictive validity to render them viable outcomes for interventional trials [23, 27].
